# Tea film formation in artificial tap water

**DOI:** 10.1039/d3sm00169e

**Published:** 2023-07-19

**Authors:** Caroline E. Giacomin, Rebecca Yun Chen, Erwin Hack, Peter Fischer

**Affiliations:** a ETH Zürich, Institute of Food Nutrition and Health Schmelzbergstrasse 7 8092 Zürich Switzerland caroline.giacomin@hest.ethz.ch peter.fischer@hest.ethz.ch; b EMPA, Transport at Nanoscale Interfaces Überlandstrasse 129 8600 Dübendorf Switzerland

## Abstract

On the surface of tea infusions, the formation of a transparent, shiny film which cracks upon disturbance can often be observed. This study aims to determine how water composition, tea varieties, and tea additives impact the formation and properties of tea film, often also called tea scum. The strength of the surface film, composed of polyphenols complexed with various ions from tap water, was investigated by interfacial rheology. Microscopy and ellipsometry were used to investigate structure and thickness of the adsorption layer, respectively. We find that green tea forms more visible layers than black tea in soft and moderate artificial tap water, but in these same waters, black tea demonstrated greater surface strength. In hard artificial tap water, green tea demonstrated greater surface strength than black. No visible layer nor surface strengthening was observed on rooibos tea. Brews in hard artificial tap water formed brittle films for green tea, fracturing at strains one order of magnitude lower than in soft or moderate. Despite large variations in film strength, black tea at all water hardness levels tested formed a film with 20 nm thickness. In black tea an increased resilience to deformation was found when adding β-casein, a protein found in milk.

## Abstract 2

On tea infusions, the formation of a transparent, shiny film which cracks upon disturbance can often be observed. The strength of this film depends on water hardness and tea variety.

## Graphical abstract

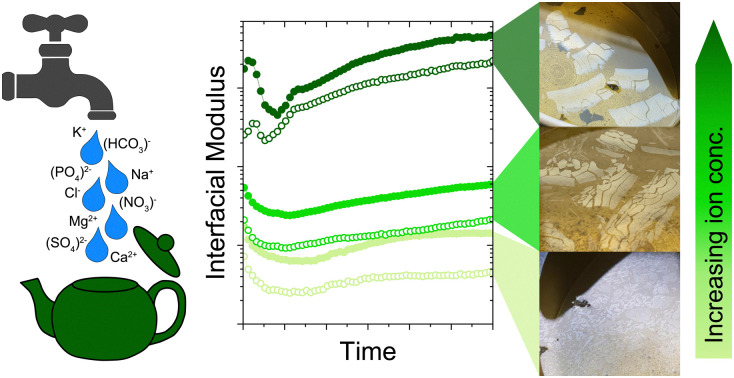

## Introduction

Tea is one of the most consumed beverages worldwide and is of great importance in various ancient and modern cultures. Tea, by definition, is only produced from leaves of *Camellia sinensis*,^1^ however, applying different processing steps results in black, green, white, oolong, or pu-erh tea,^2^ all of which have different visual and sensory properties. Although they do not meet the definition of tea,^1^ there are many other plant extract beverages colloquially referred to as “tea” such as maté, rooibos, camomile, mint, and other herbal infusions. Rooibos is brewed with leaves of *Aspalathus linearis*, traditionally grown in South Africa, while maté comes from the leaves of *Ilex paraguariensis*, native to South America.

The concentration of polyphenols varies considerably both within and between different teas. The main influences in polyphenol content include seasonal variation, leaf handling (*e.g.*, oxidation^3^), harvest method, and leaf age.^4^ Polyphenols are responsible for important tea properties *e.g.*, colour, flavour, and brightness. In addition, various health-related studies focus on the antioxidant activity of tea due to high polyphenol content.^5,6^ Further, polyphenols are known to undergo chemical complexation with ions present in the water or introduced to the brewed tea such as citrus juice, salt, or milk.^7^ By *complexation* we mean the binding of ions from the water to biopolymers present in tea. The polyphenols found in green tea are mainly catechins and the overall concentration of these polyphenols is high compared to black tea.^8^ During fermentation of tea leaves, an important step in black tea production, catechins are converted into theaflavins,^9^ which are the main polyphenol in black tea. Nevertheless, the overall polyphenol species profile is similar in both teas.^10^

### Tea additives and their effect on tea composition

Tea additives commonly used in Western countries include mainly sweeteners, lemon juice, and milk.^11,12^ While their effects on the sensory properties of tea are apparent, they can also interact with components of the tea, especially polyphenols. Antioxidant properties of black tea are enhanced by lemon juice and reduced by milk.^12^ Polyphenols, in particular those of large molecular weight (such as those in black tea), possess strong binding affinities to casein found in milk.^13^ The reduction in polyphenol health properties *via* the addition of milk is investigated and confirmed by several studies.^14–16^ In contrast, the increasing antioxidant activity caused by adding lemon juice led to contradictory results. This increase can be assigned to the degradation of complex polyphenols to simpler ones that possess higher antioxidant activity.^12^ However, larger polyphenols are also known to have stronger antioxidant activity compared to simple ones,^13^ suggesting that the degradation effect of lemon juice would lead to decreased antioxidant activity. On the other hand, acidic conditions were found to release additional polyphenols from tea leaves into the brew in studies without consideration for antioxidant activity.^17^ Consequently, the exact effect of lemon juice on polyphenol extraction remains unclear. The mineral profile of tea is also changed by lemon juice as the pH change increases the soluble fraction of iron and calcium.^18^ It has been proposed that acids liberate calcium from the calcium–polyphenol complexes, the main component of the tea film (tea scum).^19^

Adding salt to tea was mentioned in the first written description of tea preparation by Lu Yu around 760 CE,^20^ where salt addition was claimed to enhance flavour. Adding salt to tea has since been practiced in several Asian regions including Mongolia, Tibet, and Himalaya where it is known as Noon Chai, Namkeen Chai, or Jya.^21^ In addition, salted chai tea is commonly consumed by Indian tea garden workers in the Assam region.^22,23^ In India, the beverage is made from black or green tea and often consumed with milk and spices in addition to salt. Investigation of salt addition on polyphenol extraction from tea leaves showed that NaCl accelerates extraction in black tea infusions.^24^

### Influence of water hardness on tea component extraction

The composition of local tap water varies greatly depending on its origin and region. The preference for the taste of tap water has been found to correlate with mineral composition.^25^ The amount of dissolved calcium and magnesium present in water is expressed as calcium carbonate concentration (mg L^−1^) and known as water hardness.^26^ The interactions between compounds in tap water and infused tea have been studied with respect to colour, turbidity, and catechin content.^27^ Water composition has also been shown to affect sensory properties of green tea significantly and, to a lesser extent, black tea. This is mainly due to enhanced extraction rate of catechins from tea leaves infused in deionized water compared to water with higher mineral content.^28^ Furthermore, the quantity of extracted polyphenols decreases with increasing mineral concentration and complexation is the relevant mechanism regarding the influence of water composition on tea polyphenols.^29^ In the same study, calcium was found to be absorbed by tea leaves and complexed with pectin, thus decreasing the soluble calcium fraction in the tea infusion.

### Tea film formation and different influence factors

When tea is exposed to air, the phenomenon of a film forming is often observed at the air–water interface. It appears as a slightly transparent, shiny, and oily-looking film and cracks visibly into pieces upon disturbance. A series of experiments regarding the composition and the formation of this tea film have been carried out. According to a former theory, the film is a product of epidermal waxes of tea leaves released by the boiling water.^30^ However, the film has been confirmed to be composed of high molecular weight organic material with islands of calcium carbonate or bicarbonates of magnesium or manganese.^19^ Empirically, the organic component of the film was found to have 45 carbon atoms and spectroscopy confirmed hydroxyl groups, carbonyls, and unsaturated organic linkages.^19^ Another important finding was that no tea film formed on tea brewed with distilled water.^19,31–33^ Therefore, the authors concluded that ions present in tap water are crucial for the formation of this surface film.

In further studies, the presence of calcium, magnesium, and bicarbonate ions is found crucial for formation of the tea film.^19^ The organic matter most likely results from the reaction of calcium carbonate with oxidized polyphenolic compounds present at the surface of tea. Their findings also include analysis of various factors influencing the amount of tea film formed and show the film quantity is only proportional to surface area and not to the volume of the tea infusion. Tea film quantity is increased with increased surface area or increased oxygen concentration above the surface.^19^ Increasing tea leaf concentration initially increases film formation then decreases. This decrease was found to be caused by the decreasing pH resulting from the higher amount of tea leaves. It was concluded that low pH inhibits the formation of a stable detectable tea film.^31^ Different ingredients, namely lemon juice, sugar, and milk have been added to the tea infusions.^31,34^ Lemon juice lowers pH and the acid complexes with calcium in the water, thereby reducing the calcium available to complex with polyphenols. Complexation effects were also reported to be the cause of decreased film formation upon addition of sucrose.^5^ Black tea infusions containing different kinds of milk led to an increased amount and thickness of surface film. However, the properties of this film were different from the film on black tea infusion without milk. In particular, the milk tea film was found to be composed of products from the reaction of milk components and polyphenols aggregated by Ca^2+^ ions. Further work specifically looked at the production of a more efficient detergent for removal of the tea stains forming on cups or pots when the liquid level lowers due evaporation. This work found that stains were composed of oxidized polyphenols and calcium silicates instead of calcium carbonates found in tea film.^35^ Consequently, it is not simply the dried tea film collecting on tea cups but a product of further reactions.^36^

### Mechanics

Strength of the film on black tea was recently investigated by measuring the interfacial viscoelastic moduli 
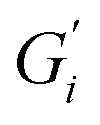
 and 
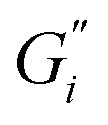
 in interfacial rheology experiments. Water hardness (defined as dissolved CaCO_3_ concentration in Milli-Q water) was found to be a significant factor for film strength.^32^ Milli-Q water is water purified with ion exchange cartridges with the resulting water having a conductivity of 18.2 MΩ cm^−1^. Tea brewed with pure Milli-Q water showed no tea film formation, while the strongest tea film formed on tea brewed with hard water containing 200 mg CaCO_3_ per L. Citric acid significantly weakens the film. Measurable surface films were not always visible. Addition of milk completely inhibited the formation of a detectable tea film with interfacial rheology, though a visible film was still observable.^32^

### Research goals

Our work investigates tea film formation by examining rheological properties of the surface film. Artificial tap water (ATW) at varied hardness levels is produced at controlled concentrations of ions present in tap water. The surface films on tea brewed with different water compositions are compared and the differences in film on black tea, green tea, and rooibos infusions are analysed alongside black tea additives, such as lemon juice and salt. In recent experiments, whole milk was added to black tea.^32^ The effect of milk as a complex mixture of proteins, lactose, and fats is difficult to view as one compound. Therefore, the isolated effect of two different milk proteins, namely β-casein and β-lactoglobulin, is studied by adding these compounds separately to black tea. Interfacial rheology, ellipsometry, and light microscopy are performed to elucidate tea film physics in ATW.

## Experimental

### Artificial tap water (ATW) preparation

To ensure consistency, artificial tap water (ATW) was produced. Important ions present in tap water include calcium, magnesium, sodium, chloride, sulfate, potassium, phosphate, and nitrate.^37,38^ The Ca^2+^ concentration thresholds for hardness^39^ were used to set values corresponding to soft water at 50 mg CaCO_3_ per L, moderately hard water at 100 mg CaCO_3_ per L, and very hard water at 200 mg CaCO_3_ per L. These concentrations will be referred to as soft, moderate, and hard, respectively, for this work. However, since not all Ca^2+^ ions were added as CaCO_3_, total Ca^2+^ concentration was set to 0.002 mol Ca^2+^ per L accordingly to correspond to the hardness bands.^39^ Hard ATW was prepared with ion ratios at prior literature values.^37^Table 1 gives the formulation and Table 2 gives the resultant ion concentrations. For moderate and soft ATW, the prepared hard ATW batch was diluted with Milli-Q water.

**Table 1:** Compounds used to prepare 1.0 L of hard ATW

Compound	Molecular weight [g mol^−1^]	mass in 1.0 L hard ATW [mg]	Purity	Manufacturer
Ca(NO_3_)_2_·4H_2_O	236.17	19.3	≥ 99.0%	Sigma Aldrich, Japan
CaCl_2_·2H_2_O	147.01	82.1	USP	Sigma Aldrich, Japan
CaCO_3_	100.09	135.9	EMSURE Ph. Eur.	Merck, Germany
KH_2_PO_4_	136.09	6.7	≥ 99.0%	Sigma Aldrich, USA
KHCO_3_	100.12	12.2	USP	Sigma Aldrich, Germany
MgSO_4_·7H_2_O	246.50	163.9	≥ 99.0%	Sigma Aldrich, Germany
Na_2_SO_4_	142.04	45.8	99.6%	Anala R NanoPur, Belgium
NaHCO_3_	84.00	37.0	100%	Sigma Aldrich, USA

**Table 2:** Ion composition of hard ATW

Ion	Concentration [mmol L^−1^]
(HCO_3_)^−^	3.28
Ca^2+^	2.00
Cl^−^	1.12
Na^+^	1.08
(SO_4_)^2−^	0.99
Mg^2+^	0.66
K^+^	0.17
(NO_3_)^−^	0.16
(PO_4_)^2−^	0.05

### Tea preparation

Black (Ceylon Black, Migros, origin: Sri Lanka) and green (Bio Grüntee Sencha, Alnatura, origin: China) tea and rooibos (Bio Rooibos, Alnatura, origin: South Africa) infusions were investigated. All experiments were performed as duplicates. Tea preparation follows ISO 3103^40^ method and ratios, with one deviation: water temperature was lowered from 100 °C to 60 °C to reduce evaporation. In 150 mL of 60 °C water, 3 g of tea leaves were secured in a stainless-steel mesh tea strainer. Loose leaf tea is used to avoid any undesired effects from tea bag materials.^41^ The infusion was agitated with the strainer to accelerate the diffusion process at 0, 3, and 6 minutes.

### Tea additives

Tea additives (NaCl, citric acid, β-casein, and β-lactoglobulin) were added to black tea to investigate their impact on film formation in ATW. The additive was added to the water just before the addition of the leaves. For salt, pure NaCl (>99% Sigma Aldrich, USA) was used. The salt concentration 8 g NaCl per L was set according to traditionally prepared salted tea.^22^ Therefore, 1.2 g NaCl was used in each brew. Citric acid (99% pure anhydrous ACROS Organics, Austria) was diluted and used to mimic adding lemon juice to tea. A concentration of 48 g citric acid per L was used.^32,42^ Into the 150 mL tea infusion, 4.5 mL of this citric acid solution was added. Isolated effects of two milk proteins were analysed: β-casein (>98%, Sigma Aldrich, USA) and β-lactoglobulin (97% provided by the Food and Bioprocess Engineering group at the Technical University of Munich^43^). Prior work added 3 mL of UHT milk (3.25% milk fat) to 100 mL tea.^32^ This same ratio was followed to calculate protein content to be added. Total protein content was 3.3% for the milk used in prior work.^32^ Cow milk protein consists of 30% β-casein and 10% β-lactoglobulin.^44,45^ Consequently, 44.6 mg β-casein and 14.9 mg β-lactoglobulin were added to the 150 mL black tea infusion.

Triplicates of the brew and measure experiment were carried out with black tea infusions containing salt. For the citric acid and milk protein brews, only one trial was performed.

### Rheological measurements

Rheological measurements were performed at the tea–air interface using a rheometer (MCR 702 TwinDrive, Anton Paar, Graz, Austria) with a bicone^46^ interfacial geometry (68.25 mm diameter, 2°) and a stainless-steel cup. Experiments were performed by lowering the bicone to the surface of freshly prepared tea immediately after removing the tea leaves at the end of a 6 minute brewing time. A changing water level would cause the bicone to lose contact with the tea film, therefore the ISO 3103 protocol^40^ temperature was lowered for this work to reduce evaporation.

The mechanical strength of the adsorbed tea film was quantified with oscillatory rheology and expressed as interfacial storage and loss moduli. An interfacial time sweep experiment was run for 2 hours with an interfacial shear strain falling within the small amplitude oscillatory shear (SAOS) region of that system and at an angular frequency of 1 rad s^−1^ where interfacial storage modulus 
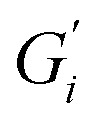
 and loss modulus 
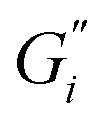
 were measured at 30 second intervals. A frequency sweep was then performed with the same SAOS region interfacial shear strain and angular frequency from 0.1–100 rad s^−1^. This was followed by an amplitude sweep with increasing shear strain from 0.01–100% at 1 rad s^−1^ to determine the interfacial shear strain breaking point of the film. Subphase viscosity was adjusted for in RheoCompass software (Anton Paar, Graz, Austria) with a constant tea subphase viscosity of 2.4 mPa s.^32,46,47^ A representative sample was chosen for each measurement.

### pH measurements

To exclude the possibility of the inhibition of tea film formation being caused by acidic conditions in the tea infusion, the pH was measured before and after the brew time as well as after rheological measurements. A pH meter (Metrohm 827 pH lab, Switzerland) was used for this purpose. Three brews were made and measured.

### Microscopic observation

The tea film formed on brews with hard ATW was visible simply by eye, but a more detailed view of film structure was observed with a microscope (DM6, Leica Microsystem, Wetzlar, Germany). Microscope slides were immersed in the tea and pulled out slowly allowing film collection. Cover slides were put on all areas with large film pieces. The edges of the cover slide were sealed using UV glue (UV repair system, BONDIC Starter+).

### Breaking and reforming

To simulate stirring tea and subsequently allowing a second film to form, rheological measurements as described in the Section above were performed and followed with a 1 minute stir with a stir bar at 300 rpm. Then, a second rheological measurement (time and amplitude sweep as described in Section 2.4) was performed.

### Ellipsometry

Measurements of film thickness and refractive index were performed using a spectroscopic ellipsometer (M-2000 VI, J. A. Woollam Co., Inc., Lincoln, United States) in the wavelength range 370–1690 nm with an integration time of 5 s. Using three incidence angles of 50°, 60° and 70° allowed us to simultaneously obtain the optical parameter and the film thickness. While the complex reflection coefficient depends on incidence angle and optical parameter, the superposition of multiple reflections from the film depends on the film thickness and is strongly wavelength and angle dependent because the optical path through the film changes with incidence angle. The film was modelled as a Cauchy material, while the brew was modelled as a Sellmeier material.

## Results

Interfacial oscillatory rheology time and amplitude sweeps for various tea preparations are found to develop storage and loss moduli plateaus indicating the formation of viscoelastic interfacial adsorption layers. The effect of leaves, add-ins, and pH are discussed.

### Rheology of green and black tea films

Interfacial time sweep experiments were performed to compare the film formation process of black tea, green tea, and rooibos infusions brewed with ATW of various hardness levels. Furthermore, time sweeps were conducted with black tea containing additives, namely lemon juice, salt, or milk proteins. Initially, to prevent surface disruption caused by removal of the metal mesh sphere holding the tea leaves, experiments were run with tea leaves in the brew for the entire duration of the rheological test. However, preliminary tests showed that surface disruption did not affect results on film strength, therefore all results presented had tea leaves removed at the 6 minute mark defined in ISO 3103.

**Fig. 1:**
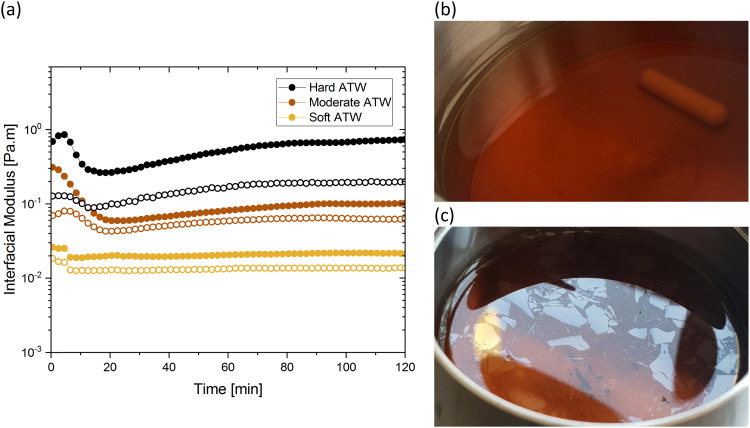
(a) Time sweeps showing storage 
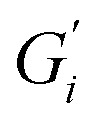
 and loss 
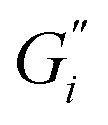
 moduli, closed and open circles respectively, for 3 g of black tea brewed in 150 mL of hard, moderate, and soft ATW at 60 °C for the 6 minute brew. Photographs of film fragments after geometry removal in black tea brewed with (b) soft ATW and (c) hard ATW.

Fig. 1 shows the viscoelastic moduli for black tea prepared in hard, moderate, and soft ATW. Moduli were stabilized after the first hour of the experiment. Milli-Q water showed no sign of measurable tea surface film. The film strengths measured, expressed as 
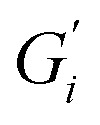
 and 
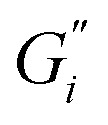
, was highest for hard ATW at 0.7 Pa m and 0.2 Pa m for 
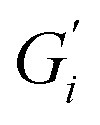
 and 
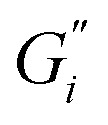
, respectively, and lowest for soft ATW at 0.02 Pa m and 0.01 Pa m for 
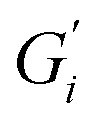
 and 
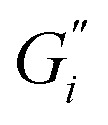
, respectively. The moduli of the moderate ATW brew were stabilized at 0.1 and 0.06 Pa m for 
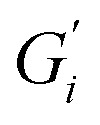
 and 
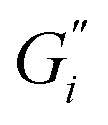
, respectively.

**Fig. 2:**
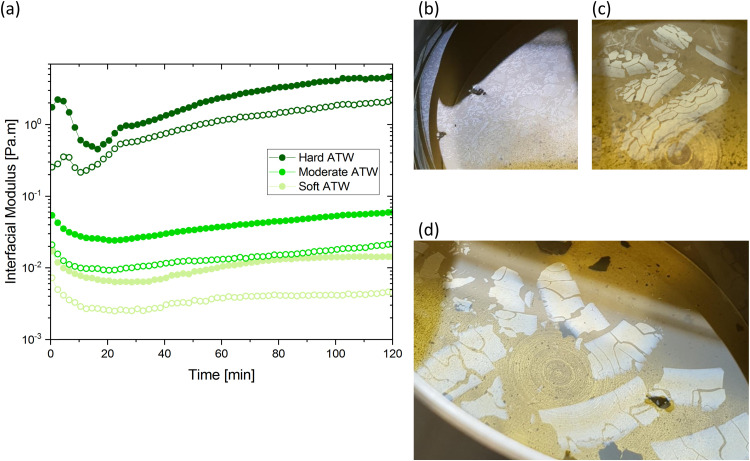
(a) Time sweeps showing storage 
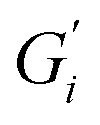
 and loss 
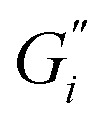
 moduli, closed and open circles respectively, for 3 g of green tea brewed in 150 mL of hard, moderate, and soft ATW at 60 °C for the 6 minute brew. Visible tea surface film broken into pieces on green tea brewed with soft ATW (b), moderate ATW (c), and hard ATW (d).

The results in Fig. 1a are consistent with visually perceived findings for hard ATW brew (Fig. 1b and c). For black tea, elastic behaviour always dominated viscous behaviour. Fig. 2a shows the time sweep moduli of green tea surface film. These films established plateaus more quickly than in black tea, but the plateaus were less stable. Surface films were observable by eye for soft, moderate, and hard ATW green brews (Fig. 2b–d), whereas the black tea surface films were visually absent for soft (Fig. 1b) and medium ATW.

Comparing these, the interfacial moduli for soft ATW green tea and black tea were on the same order of magnitude but green tea displayed a larger disparity between the storage and loss moduli. Green tea brews prepared with moderate ATW reached lower moduli values than the moderate ATW black and did not form a real plateau during the measurement duration. An initial stabilization period of 20–40 minutes is present in all time sweeps and is possibly due to temperature equilibration.

**Fig. 3:**
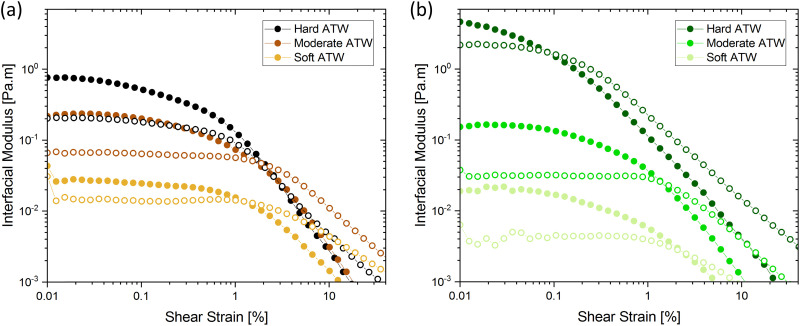
Amplitude sweeps showing storage 
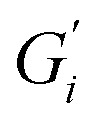
 and loss 
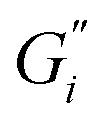
 moduli, closed and open circles respectively, for 3 g of tea brewed in 150 mL of hard, moderate, and soft ATW at 60 °C for the 6 minute brew: (a) black tea and (b) green tea.

In amplitude sweep experiments (Fig. 3), the deformation at the moduli crossover point (the intersection of storage and loss moduli, where the film cracks) is considered the dynamic yield stress. Amplitude sweeps for black tea (Fig. 3a) display elastic properties dominate in all ATW tested. The modulus at which the dynamic yield point occurs decreases with decreasing water hardness. The shear strain for the dynamic yield point does not change with water hardness. The amplitude sweep for green tea (Fig. 3b) shows elastic properties dominating in all ATW tested. For green tea brews, both the moduli and shear strain of the dynamic yield point decrease with decreasing water hardness. This allows the conclusion that green tea is more susceptible to forming strong films with lower ion concentrations.

**Fig. 4:**
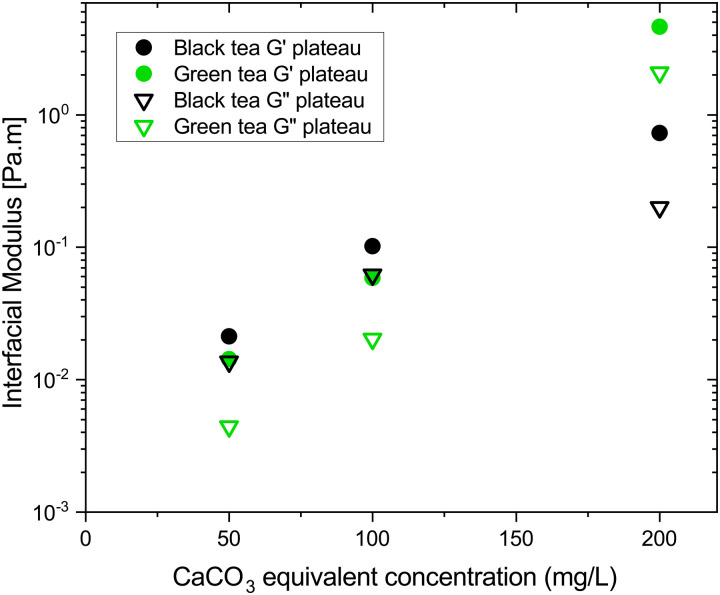
Plateau values of storage 
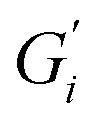
 and loss 
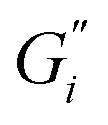
 moduli for black (black symbols) and green (green symbols) tea films formed in soft, moderate, and hard ATW plotted against CaCO_3_ equivalent concentration. Storage modulus is represented with solid symbols and loss with open symbols.

Fig. 4 summarizes the storage and loss moduli plateaus obtained in Fig. 1a and 2a plotted as single points for each hardness level tested. Comparison of green and black tea results indicate the moduli of green tea films are more sensitive to water hardness. In moderate and soft ATW, the moduli of black tea exceed the moduli of green tea. However, in the hardest water, the black tea brews exhibit lower moduli than green tea brews. When this is compared to previous literature, where the same black tea was brewed with only CaCO_3_,^32^ the more complex environment of ATW yields moduli plateaus an order of magnitude lower. Green tea does not have a literature comparison.

**Fig. 5:**
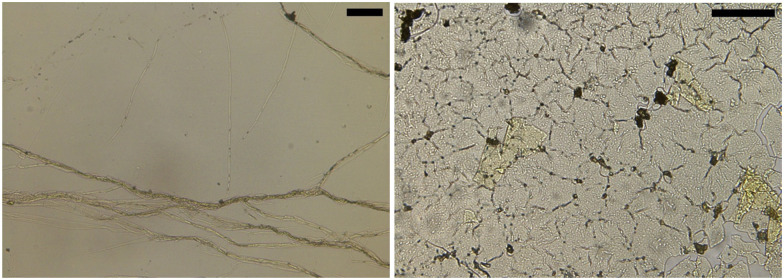
Microscopy for black (left image) and green tea (right image) films from hard ATW brews. Scale bars: 100 μm.

The rheological difference we observe was investigated with microscopy of hard ATW brews (Fig. 5). Island size and shape deviate greatly. The hard ATW black tea film has longer and straighter fracture lines which rarely terminate except at junctions with other fractures. The green tea film has more organically curved fractures and these fractures generally self-terminate.

### Influence of citric acid, salt, and milk

**Fig. 6:**
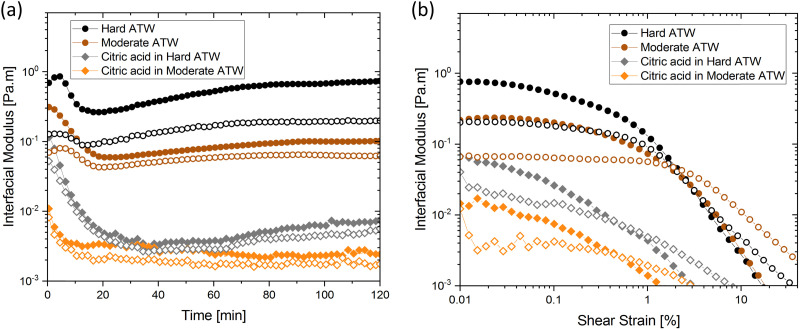
(a) Time and (b) amplitude sweep for hard and moderate ATW black tea brews when citric acid is added. Circles represent the ATW brews, and diamonds indicate added citric acid. Solid symbols represent storage modulus and open symbols represent loss modulus. All teas brewed with 150 mL ATW and 3 g tea leaves. Citric acid, 4.5 mL, added to the trials indicated.

Citric acid addition to black tea in hard and moderate ATW brews reduced the modulus plateaus to below 10^−2^ Pa m (Fig. 6a). No surface film was visible on either of these two tea infusions, however surface strengthening still occurred. Citric acid added to the soft ATW brew developed no detectable film. Elastic behaviour dominates over viscous behaviour, as in the corresponding measurements for pure black tea, graphed alongside the citric acid results in Fig. 6a and b. For brews with added citric acid the moduli both yielded at an amplitude of 0.5% (Fig. 6b), while in plain brews the yielding occurred at 2.0%.

**Fig. 7:**
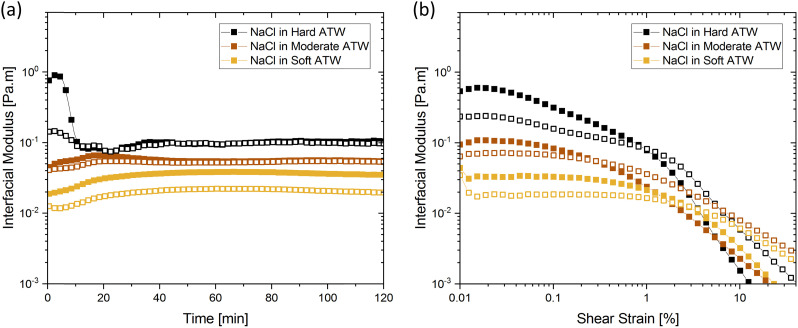
(a) Time and (b) amplitude sweeps for hard, moderate, and soft ATW black tea brews when NaCl is added. Solid symbols represent storage modulus and open symbols represent loss modulus. Brews of 150 mL with 3 g black tea leaves have 1.2 g NaCl added and dissolved before the 6 minute brew time.

Black tea containing additional salt (NaCl), brewed with soft, moderate, and hard ATW was also investigated in time sweep experiments. The results shown in Fig. 7 differed from the results of plain black tea by: (i) reducing the deviation between 
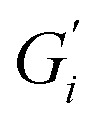
 and 
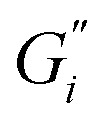
 for moderately hard ATW, (ii) decreasing the plateau for hard ATW by nearly one order of magnitude, and (iii) slightly increasing the plateaus for soft ATW. These deviations, in aggregate, reduced the differences normally observed by the water hardness deviations and therefore suggest that salt addition brings many water compositions to a more neutral, standard result. NaCl is commonly used in water softeners,^48^ so it is unsurprising that it lowers the hardest water results.

**Fig. 8:**
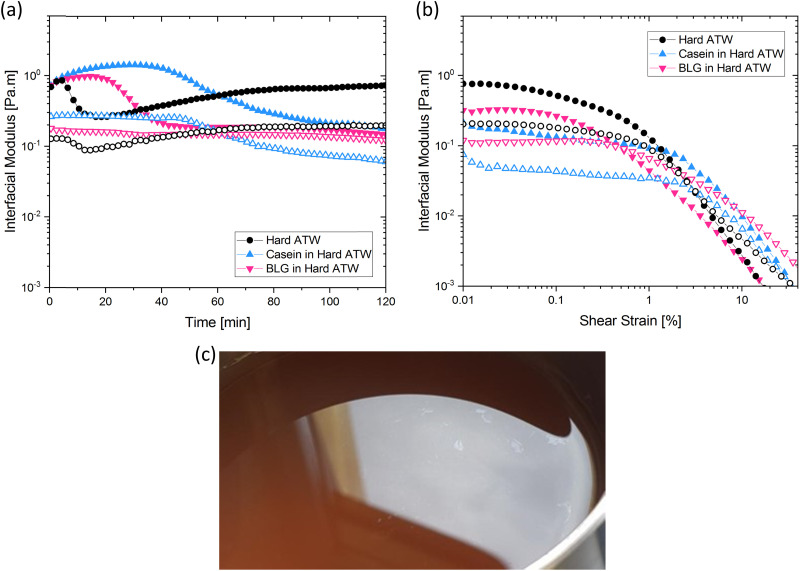
(a) Time and (b) amplitude sweeps for hard ATW black tea brews when β-casein or β-lactoglobulin is added. Solid symbols represent storage modulus and open symbols represent loss modulus. Brews of 150 mL with 3 g black tea leaves have proteins added before the 6 minute brew time. (c) Small pieces of visible black tea film on a matte film on black tea brewed with hard ATW containing β-casein.

In prior work, milk was found to have no film strength measurable^32^ despite milk forming a visible film. This film is also known to be chemically different than non-milk tea films.^34^ Greatly enhanced amount and thickness of surface films on black tea with milk have been observed.^34,49^ To isolate the milk component reducing the measurable signal, β-casein and β-lactoglobulin are added separately to black tea brewed with hard ATW and shown in Fig. 8a and b. The addition of either milk protein results in lower values for 
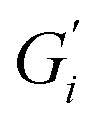
 and 
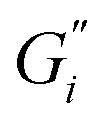
 compared to black tea prepared with hard ATW. At the end of the experiment, the values differed by almost one order of magnitude with 
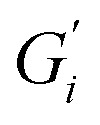
 and 
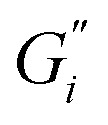
 slightly below 0.2 Pa m for the black tea containing β-lactoglobulin. 
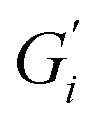
 of the black tea containing β-casein reached 0.2 Pa m whereas 
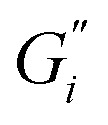
 was at 0.06 Pa m. Interfacial moduli values for black tea containing β-lactoglobulin reached a plateau after approximately 60 minutes, akin to black tea without proteins. In contrast, the tea infusion with β-casein did not reach an equilibrium.

Different surface phenomena could be visually observed in black tea upon the addition of milk proteins compared to the tea film forming on pure black tea. The effect of β-casein can be seen in Fig. 8c. Tea infusion bulk appeared slightly cloudy, and a matte film covered the surface apart from a few small pieces of shiny tea film. The effect of β-lactoglobulin is not pictured as no visible film was present.

### Stir and reform

**Fig. 9:**
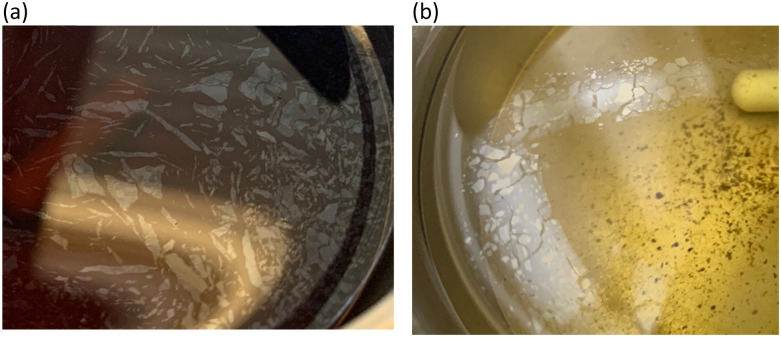
Films observed after second rheological protocol run for hard ATW brews for black (a) and green (b) tea. Brews are made with 3 g leaves and 150 mL of hard ATW, a time sweep and amplitude sweep are run, a 1 minute stir is performed at 300 rpm, and the rheological sweeps are repeated. Photos were taken after bicone is removed the second time.

For stirred and reformed brews, the surface film self-heals into a film with interfacial moduli one order of magnitude higher than the original film. This holds for black and green brews in both hard and moderate ATW. One can observe visually, in Fig. 9, that the new film that forms contains fragments of the first film.

### Influence of pH values on film strengths

**Fig. 10:**
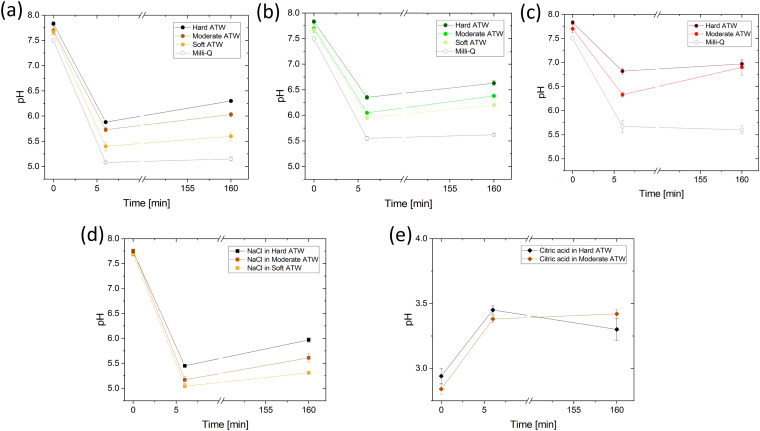
pH of (a) black tea, (b) green tea, and (c) rooibos tea brewed with hard, moderate, and soft ATW and Milli-Q water. The pH of black tea with addition of (d) NaCl and (e) citric acid are shown with hard, moderate, and soft ATW. Plots (a)–(e) are measured at the beginning and end of the experiment and after the 6 minute brew.

The presented effect of citric acid and salt can be viewed in light of the induced pH changes and are summarized in the following. First, the pH of the different ATWs was measured for all infusions as a function of time without addition of citric acid or salt. Fig. 10 shows the overall acidity decreases during the first 6 minute brew, and then mildly increases over the 2 hour rheological experiment. This increase suggests that acidic components were neutralized by reacting with other tea compounds or *via* bicarbonates of calcium reacting with excess H^+^ in the acidic environment and producing CaCO_3_ and CO_2_ gas.^50^ For all tea varieties, increasing water hardness corresponds with a pH increase. Knowing how Ca^2+^ and CO_3_^2−^ behave in various pH environments, this is predictable.^50^ For NaCl addition, the pH is lowered and the differences in pH between hardness levels is buffered by the NaCl when Fig. 10d is compared to Fig. 10a. Acidity is of interest as when citric acid lowers the pH of black tea brews (Fig. 10e), the film strength is vastly reduced (see Fig. 6). The pH was measured to confirm the rooibos infusions were not lacking film due to pH reasons, but rather due to polyphenol composition of rooibos differing from teas of *Camellia sinensis* (black and green teas).

### Ellipsometry

**Fig. 11:**
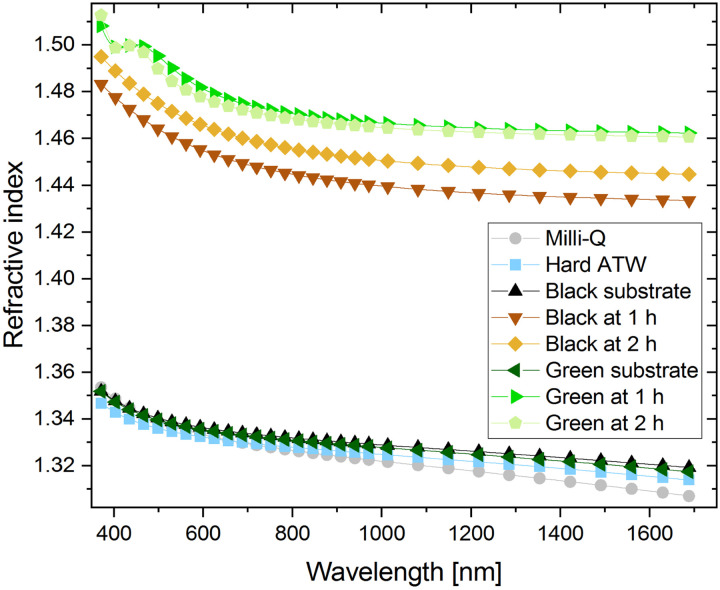
Refractive indices over the wavelength range 370–1690 nm are shown for Milli-Q, Hard ATW, and tea brews prepared with hard ATW. The tea brews were measured at 1 and 2 hours and substrate measurements come from wiping the film off after the 2 hour measurement and the cleared surface being measured.

As thickness and refractive index are interrelated, refractive indices of various liquid surfaces over a range of wavelengths are shown in Fig. 11. Hard ATW, Milli-Q, and both substrates, green and black tea prepared with hard ATW, demonstrated similar refractive indices and therefore the brew was modelled as a Sellmeier material. At both 1 and 2 hours after brewing, tea films for black and green hard ATW brews demonstrated higher refractive indices.

Black teas brewed with hard, moderate, and soft ATW, despite their rheological differences, were found to have a similar film thickness of 17 ± 2 nm after 1 hour and 20 ± 2 nm after 2 hours. When black tea is brewed at double strength (twice the tea leaves/volume as ISO 3103^40^), a 35% increase in film thickness was observed. These findings suggest the thickness of the film had no bearing on the interfacial rheology and the strength was therefore based on film density or film structure. Earlier work showed that water hardness affected the mass per area of the film generated,^31^ so the density of these less rheological strong films must have been reduced since thickness remains constant.

## Discussion

The tea film is known to consist mainly of polyphenols and calcium carbonate.^19^ Therefore, free calcium and bicarbonate ions are crucial for the occurrence of tea film formation. It is reasonable that the strength of the film increased with increasing water hardness, however there is a counteracting effect of water composition on tea film formation. The extraction of polyphenols is enhanced in water containing fewer ions.^27–29^ Since oxidized polyphenols and calcium carbonate are the main components of the film, the amount of these compounds will affect the extent to which tea film formation occurs and, consequently, impact the surface film properties. Since film strength increased with increasing water hardness, despite the known decreased polyphenol extraction, it can be concluded that the quantity of polyphenols was not a limiting factor in the tea film formation process in black tea. However, the quantity of polyphenols needed for the formation of tea film of a certain strength remains unknown.

The lack of measurable tea film strength for rooibos infusions confirms surface strengthening has not occurred. When acidic conditions were considered, rooibos was still found to be an anomaly as the rooibos was found to be the least acidic of all three investigated brews. Therefore, the lack of film in rooibos infusions and the measurable surface film on black and green tea can be attributed to the differences in the polyphenol profile of rooibos compared to black and green tea. While black tea and green tea show differences in the ratios of present polyphenols, the polyphenol species present are similar, rooibos was found to contain different polyphenols.^5^ While the total amount of polyphenols was considerably higher in rooibos than in green and black tea,^51^ rooibos is known to sometimes lack the polyphenol rutin.^52,53^ Additionally in rooibos, the polyphenols catechin and theaflavin have concentrations an order of magnitude lower than in green or black tea.^53^

## Conclusion

Water hardness is the main influence on the interfacial strength of tea films for green and black tea. Strength of the tea films formed increased with increasing water hardness. Polyphenol concentration in tea brewed with soft water is not the limiting factor for tea film strength. Dependence of strength on water hardness can be explained by increased concentration of Ca^2+^.

The surface film on black tea appears more brittle and shows dominating elastic behaviour. In contrast, viscous behaviour is dominant in the film forming on the hardest ATW green teas which results in decreased brittleness. The structural differences between these surface films are associated with the higher polyphenol content of green tea and to the polyphenol species present. Stirring and reforming tea films, for both green and black, results in the second film having higher storage and loss moduli.

The addition of lemon juice decreases the strength of the surface film on black tea to considerably lower interfacial moduli values. The effect of salt (NaCl) addition to black tea on the film strength indicates that salt reduces the effect of water hardness on tea film formation in black tea. Isolated effects of β-casein or β-lactoglobulin on film strength showed a decrease in the film strength caused by tea component interactions with these milk proteins. The interaction of β-casein and Ca^2+^ and polyphenols leads to the formation of a structurally different, more resilient tea surface film, which was resistant to high deformation. However, such a film does not reach an equilibrium.

No surface strengthening phenomena occur on rooibos infusions. As polyphenols of rooibos differ greatly from the polyphenol profile of black and green tea, the precise polyphenol profile of *Camellia sinensis* is critical.

The findings will be of interest to producers of instant tea beverages in which film formation might be undesirable. Further, the knowledge might be useful for the treatment of dried tea leaves to limit film formation in the brewing process, thus decreasing staining in industrial tea brewing equipment.

## Author contributions

Caroline E. Giacomin: conceptualization; data curation; formal analysis; investigation; methodology; validation; visualization; writing – original draft; writing – review & editing. Rebecca Yun Chen: data curation; formal analysis; investigation; methodology; validation; visualization; writing – original draft; writing – review & editing. Erwin Hack: data curation; formal analysis; writing – original draft; writing – review & editing; investigation; methodology; resources. Peter Fischer: conceptualization; data curation; project administration; resources; supervision; writing – review & editing.

## Conflicts of interest

The authors declare no conflict of interest.

## Supplementary Material
